# Current status of sevoflurane anesthesia in association with microglia inflammation and neurodegenerative diseases

**DOI:** 10.1002/ibra.12021

**Published:** 2022-02-23

**Authors:** Yan‐Li Huang, Zhao‐Qiong Zhu

**Affiliations:** ^1^ Department of Anesthesiology The Affiliated Hospital of Zunyi Medical University Zunyi Gui Zhou China

**Keywords:** inflammatory factor, inflammatory signaling pathway, microglia, neurodegenerative diseases, sevoflurane

## Abstract

Sevoflurane is one of the most commonly used volatile anesthetics in clinical practice and is often used in pediatric anesthesia and intraoperative maintenance. Microglia exist in the central nervous system and are innate immune cells in the central nervous system. Under external stimulation, microglia are divided into two phenotypes: proinflammatory (M1 type) and anti‐inflammatory (M2 type), maintaining the stability of the central nervous system through induction, housekeeping, and defense functions. Sevoflurane can activate microglia, increase the expression of inflammatory factors through various inflammatory signaling pathways, release inflammatory mediators to cause oxidative stress, damage nerve tissues, and eventually develop into neurodegenerative diseases. In this article, the relationship between sevoflurane anesthesia and microglia inflammation expression and the occurrence of neurodegenerative diseases is reviewed as follows.

## Graphical abstract

Sevoflurane activates microglia and releases inflammatory cytokines (such as IL‐1β, IL‐6, and TNF‐α) through inflammatory signaling pathways, such as Notch, Toll‐like, AMPK, and NF‐κB or reduce the release of inflammatory factors (such as AMPK signaling pathway). Overexpression of inflammatory factors or dysfunction of microglia can eventually lead to neurodegenerative diseases (such as Alzheimer's disease, postoperative cognitive dysfunction, multiple sclerosis, and Rett syndrome).

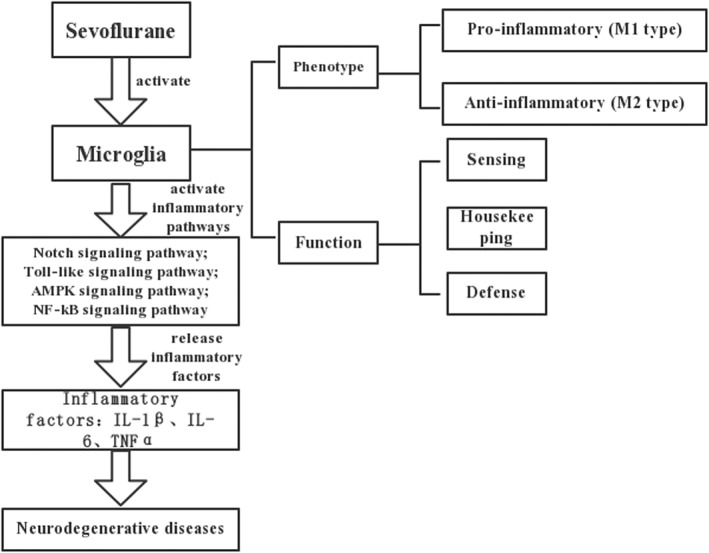

## 1 INTRODUCTION

Microglia originated from the yolk sac of the embryo and existed in the central nervous system for a long time. Microglia are the most important immune defense line in the central nervous system, participating in the balance in vivo and the host's defense against pathogens and central nervous system disorders, and promoting brain development under normal conditions.^1^ Sevoflurane is often used for anesthesia in pediatric or adult outpatient minor operations or exploratory operations because of its rapid induction, no irritating taste, easy control of anesthesia depth, and easy awakening. Studies have shown that sevoflurane impairs the consolidation of long‐term emotional memories, resulting in neurobehavioral deficits that may persist into adulthood after exposure to sevoflurane in the neonatal period. The use of sevoflurane in early childhood may lead to cognitive impairment later in life.^2^, ^3^ The effects of sevoflurane on cognitive impairment and neuronal apoptosis are significantly increased when exposed to high concentrations for a long time. The mechanism of sevoflurane can increase the level of inflammatory factors of microglia cells by enhancing the transcriptional activity of NF‐κB, leading to the occurrence of neuroinflammation and eventually neurodegenerative diseases.^4^, ^5^ In this paper, the research status of sevoflurane on the expression of microglial inflammation and associated degenerative diseases is reviewed as follows.

## 2 MICROGLIA

Glial cells are divided into astrocytes, oligodendrocytes, microglia, and ependymal cells. Microglia account for 10%–15% of the total number of glial cells in the human brain and are the main neuroimmune cells.^1^ Microglia are the main neuroimmune cells. In steady‐state, microglia coordinate the development of neurons and provide nutritional support to surrounding cells. In response to tissue injury or infection, microglia are activated and release inflammatory factors, reactive oxygen species (ROS), and nitric oxide (NO).^6^ Resolution of inflammation, tissue repair, and remodeling is promoted through phagocytosis and neurotrophic support.^7^ Therefore, the maintenance of microglia tissue homeostasis in the central nervous system mainly depends on microglial phenotype, activation status, and function.

### 2.1 Different phenotypes of microglia and their activation status

In normal brain tissue, microglia is in a resting state, which can be activated in infection, trauma, and neurodegenerative diseases. During activation, the “amoeba‐like” transformation of microglia causes it to produce and secrete cytokines, chemokines, and other immune mediators to respond to this invasion.^8^ According to the different phenotypes of microglia activation, they can be divided into two opposite activation phenotypes: proinflammatory (M1 type) and anti‐inflammatory (M2 type), thus microglia have cytotoxic or neuroprotective effects.

According to the activated environment or stimulating factors, microglia have three states of "classical activation," "alternative activation," and "acquired inactivation."^9^ M1 type associated with classical activation, which increases proinflammatory cytokines, such as tumor necrosis factor‐α (TNF‐α), interleukin‐1β (IL‐1β), superoxide, NO, and ROS. The chronic activation of microglia leads to excessive accumulation of these factors and neuronal damage, such as Alzheimer's disease (AD), Parkinson's disease (PD), and amyotrophic lateral sclerosis (ALS). In contrast, the M2 type increases anti‐inflammatory cytokines and is associated with alternative activation and acquired inactivation. Alternative activation is used only to deal with the inflammatory state of IL‐4 or IL‐13 and secretes anti‐inflammatory cytokines such as interleukin‐10 (IL‐10), which are involved in phagocytosis of cell debris and wound healing.^1^ Acquired activation is another state that alleviates acute inflammation, and is mainly caused by apoptotic cells, anti‐inflammatory cytokines such as IL‐10 and transforming growth factor‐β (TGF‐β).^10^ Therefore, when the brain is invaded by external substances, the microglia are the first to have the M1 phenotype, and to restore tissue homeostasis, they need to enter the anti‐inflammatory period, otherwise, the long‐term uncontrolled production of proinflammatory cytokines, NO, and ROS will lead to irreversible damage and even death of cells and tissues. Studies have shown that the activated M1/M2 phenotypic transformation can be determined according to the development stage and severity of the disease.^11^

### 2.2 Function of microglia

#### 2.2.1 Sensing

Microglia form a network in the brain which can be the product of nearly a hundred genes that sense changes in the peripheral nervous environment.^12^ Sensor messenger RNAs (mRNAs) were uniformly expressed in microglia distributed throughout the nervous system, suggesting that microglia were capable of performing their sensing functions. Sensing is the first requirement for microglia to perform other functions.^13^

#### 2.2.2 Housekeeping

Namely, physiological housekeeping function. Synaptic remodeling, migration to sites of neuron death to phagocytose dead cells or debris, and balance maintenance are major tasks of housekeeping function. The genes involved in the management of microglia include genes encoding chemokines and chemokine receptors, genes for phagocytosis, and genes for synaptic pruning in neurodegeneration, which play important housekeeping roles in the development,maintenance of normal functon, senescence and injury of the central nervous system.^9^

#### 2.2.3 Prevent self‐injury and nonself stimulation

Microglia mediate host defense against external stimuli, such as pathogens and central nervous system tumors. In response to these stimuli, microglia can trigger an inflammatory response, producing inflammatory cytokines and chemokines that recruit more cells to migrate to the lesion and induce them to clear harmful substances. It plays a defensive function by mediating inflammation and protects the body from harmful stimuli of self and nonself. But persistent neuroinflammation induces neurotoxicity and leads to neurodegeneration.^14^

Microglia cells are unable to play these protective functions due to damage of sensing or housekeeping function or maladjustment of defense function for any reason, which may damage or kill neurons and induce neurodegenerative diseases. Neurodegenerative diseases, such as AD, PD, ALS, Huntington's disease, and frontotemporal dementia may all be associated with disruption of microglia function.

### 2.3 The inflammatory body NLRP3 in microglia

Abnormal activation of inflammasome bodies in NOD‐, LRR‐, and pyrin domain‐containing protein 3 (NLRP3) microglia is also involved in the occurrence and development of various neurological diseases.^15^ The activation of the NLRP3 inflammasome mediates the maturation, secretion, and release of proinflammatory factors, such as IL‐1β and IL‐18, and initiates the inflammatory cascade.^16^, ^17^ For example, chronic stress induced by overactivated NLRP3 inflammasome induces depression in rats.^18^ Activation of mouse microglia and maturation of IL‐1β secretion mediated by NLRP3 inflammasome are associated with the development and progression of AD in animal models.^19^ Studies have shown that overactivated NLRP3 inflammasomes in microglia and IL‐1β‐mediated inflammation are involved in hypoxemia and inhaled anesthetic‐induced cognitive dysfunction in adult rats.^20^, ^21^ It can be seen that after sevoflurane activates microglia, the increase of inflammatory factors expressed by microglia may be an important cause of neurodegenerative diseases.

## 3 SEVOFLURANE ACTIVATES MICROGLIA‐RELATED INFLAMMATORY PATHWAYS AND INCREASES THE EXPRESSION OF INFLAMMATORY FACTORS

Studies have shown that sevoflurane can accelerate the migration of microglia, promote their activation and enhance their phagocytic function.^22^ Treatment with 4% sevoflurane for 6 h has been reported to increase IL‐6 in microglia via the NF‐κB pathway.^23^ Sevoflurane can further activate microglia by activating the NF‐κB signaling pathway, and microglia can increase or decrease the expression of inflammatory factors (such as IL‐1β, IL‐6, and TNF‐α) through Notch‐signaling pathway,^24^ Toll‐like signaling pathway,^25^ AMPK signaling pathway, NF‐κB signaling pathway,^26^ and other inflammatory signaling pathways. The neuroinflammation caused by the continuous increase of inflammatory cytokines may be related to the occurrence of degenerative diseases.

### 3.1 Notch signaling pathway

The Notch signaling pathway is very important in signal transduction between two adjacent microglia. In mammals, there are four types of Notch receptors (Notch 1, 2, 3, and 4), and five types of ligands (Jagged 1 and 2, Delta 1, 3, and 4). The receptor and ligand interact through the DSL domain located at the N‐terminus of the protein. After effectively binding to the ligand, the Notch receptor releases the intracellular region (NICD) with nuclear localization signal and transfers to the nucleus to bind with CSL to regulate the expression of downstream genes. In the central nervous system, activated microglia upregulated the expression of the Notch1 receptor and the ligand Jagged1, and increased the secretion of proinflammatory factors.^27^

### 3.2 Toll‐like signaling pathway

Toll‐like receptors belong to Ⅰ type transmembrane receptors, which are composed of extracellular domain, transmembrane domain, and intracellular Toll/IL‐1 (TIR) domain. Ten types of TLR receptors have been identified (TLR1‐10), each of which recognizes a different stimulus. The intracellular domain of TLR binds to the TIR domain‐containing connector molecules, which can activate the signaling pathway. TLR4 was highly expressed in postoperative brain microglia. TLR4 can mediate signal transduction through both dependent and nondependent pathways of Myd88. The former can lead to the expression of proinflammatory factors. Once TLR4 is activated by the ligand, the adapter protein MyD88 binds to the receptor and induces autophosphorylation of interleukin‐1 receptor‐associated kinase (IRAK). IRAK then separates itself from the receptor complex and binds to TRAF6. This subsequently activates two major inflammatory pathways: NF‐κB and mitogen‐activated protein kinase. Therefore, the expression of proinflammatory cytokines and chemokines was significantly increased.^28^ Studies have shown that the mRNA expression of TLR4 and NF‐κB is up‐regulated and the secretion of inflammatory cytokines increased in the model of cerebral hemorrhage in mice. However, Dahhuang Quyu Pills could regulate the M1/M2 polarization of microglia cells by inhibiting the activation of TLR4 signals, thus producing anti‐inflammatory effects.^29^ It can be proved that the loss of TLR4 function may play a neuroprotective role.

### 3.3 AMPK signaling pathway

AMPK is a heterotrimer composed of adenosine monophosphate‐activated protein kinase, which is an important receptor for regulating cellular energy homeostasis and metabolic pathways. AMPK can inhibit the M1 type of microglia cells, promote the M2 type expression, and reduce inflammatory injury. AMPK can be activated by liver kinase B1 and calcium/calmodulin‐dependent protein kinase kinase β (CaMKKβ), and the anti‐inflammatory effect of microglia is enhanced by the activation of CaMKKβ. Therefore, the CaMKKβ–AMPK signaling pathway is an important pathway to increase anti‐inflammatory factors.^30^ In addition, AMPK is an upstream kinase that mediates the activation of peroxisome proliferator‐activated receptor‐gamma (PPARγ). PPARγ is related to cognitive function and is a key regulator of inflammatory response. It may exist in many types of brain cells, including microglia, astrocytes, and neurons, activation of PPARγ in microglia lead to reduced activation of these cells, which in turn reduces production of pro‐inflammatory cytokines. Recent studies have shown that by downregulating hippocampal PPARγ, sevoflurane can increase neuroinflammation in mice and rats, as well as impair cognitive function and impair learning and memory.^31^ Therefore, it can be seen that the AMPK signaling pathway cannot be ignored in microglial activation and AMPK may be used as a therapeutic target in neuro anti‐inflammatory therapy.

### 3.4 NF‐κB signaling pathway

NF‐κB belongs to a family of inducible dimer transcription factors that recognize specific DNA sequences and regulate target genes associated with inflammation, stress, and more, and therefore, plays a key role in inflammation and immunity. NF‐κB exists in the cytoplasm in the form of p65 and p50 heterodimers. After activation, NF‐κB enters the nucleus and promotes the activation of transcription, such as IL‐1β, IL‐6, TNF‐α, cyclooxygenase 2 (COX2), and nitric oxide synthase (NOS). It also induces the secretion of ROS, which is one of the most important pathways in neuroinflammation medieated pathologic processes.^23^ In addition, sevoflurane increases cytoplasmic calcium by activating inositol 1,4,5‐triphosphate receptors, leading to the abnormal release of Ca^2+^ from the endoplasmic reticulum.^32^ Ca^2+^ elevation can induce the activation of the NF‐κB signaling pathway, which promotes the release of microglial inflammatory factors and causes neuroinflammation.^33^

The participation of microglia in these signaling pathways does not exist in isolation. The interaction of multiple inflammatory pathways leads to the release of a large number of inflammatory factors and finally causes neuroinflammation to act on the central nervous system and damage cognitive function.

## 4 SEVOFLURANE AND NEURODEGENERATIVE DISEASES

### 4.1 Sevoflurane impairs memory function

Research has shown that the consolidation of emotional memories is linked to communication between the hippocampus and the basolateral amygdala.^34^ Sevoflurane can impair the consolidation of long‐term emotional memory. The effect of emotional arousal on memory through the interaction between the amygdala and other regions is considered to be a neurobiological mechanism. In the study of the human body, in a group of volunteers were able to block the minimum memory advantages associated with arousal sevoflurane dose, phenomenon of type by positron emission computed tomography technology measure the utilization rate of cerebral glucose metabolism rate, statistical parameter mapping(SPM), a structural equation model (SEqM) analysis of sevoflurane regional specificity effect and interaction between brain areas. It was found that 0.25% sevoflurane inhibited effective connections between the hippocampus and the amygdala, thus preventing memory promotion associated with emotional arousal. It can be seen that sevoflurane can block memories related to emotional arousal, and even impair the improvement of human emotional memory.^2^ Working memory is a kind of important cognitive function, which refers to the temporary storage and operation of information necessary for the completion of complex cognitive tasks. Working memory is supported by the prefrontal cortex. In human subjects, functional magnetic resonance imaging experiments showed that the prefrontal cortex was simultaneously activated during working memory tasks.^35^ Cognitive process is completed through functional connectivity of neurons in the brain network, and the level of functional connectivity between working memory and excitatory neurons in the prefrontal cortex is also closely related. Sevoflurane decreased functional connectivity between excitatory neurons in the prefrontal cortex and impaired working memory, and the degree of impairment was related to sevoflurane concentration, anesthesia exposure time, and age of the patients. The higher the sevoflurane concentration, the longer the anesthetic exposure time, and the older the patient, the greater the degree of injury.^36^

### 4.2 Sevoflurane and AD

Sevoflurane has been shown to be associated with AD, which is primarily the deposition of β‐amyloid and intracellular hyperphosphorylated tau protein, which is neurotoxic and triggers neurodegenerative processes in the brain. Therefore, β‐amyloid and Tau deposition are the core mechanisms that drive AD.^37^ After sevoflurane exposure, β amyloid is deposited in cerebrovascular cells,^38^ and factor associated suicide (FAS) binds to factor associated suicide ligand (FASL). Activation of downstream cysteine proteinase‐3 (caspase‐3) protein mediates the formation of apoptotic bodies and promotes cell recognition and phagocytosis.^39^ Therefore, the neuronal apoptosis effect of the FAS/FASL apoptosis gene leading to neuron loss may be one of the reasons for the occurrence of AD.^40^ As a housekeeper, microglia can phagocytize and remove β‐amyloid protein aggregation to prevent AD, but as a result of aging, the function of microglia to prevent AD decreases. With the accumulation of toxic amyloid types, microglia are induced to secrete neurotoxic cytokines to damage neurons, resulting in neurodegeneration.^41^ In addition, sevoflurane can increase the content of tau protein, which activates the NF‐κB signaling pathway through the neuron–ev‐microglia pathway or the neuron–non‐ev‐microglia pathway, resulting in increased IL‐6 content and cognitive impairment.^42^ Therefore, sevoflurane can lead to the deposition of beta‐amyloid and tau protein. When the housekeeping function of microglia is not enough to phagocytize and remove the accumulated toxic amyloid, it may subsequently lead to the occurrence of AD.

### 4.3 Sevoflurane and postoperative cognitive dysfunction (POCD)

Post‐operative cognitive dysfunction (POCD) refers to the changes of mental activities, personality, social activities and cognitive ability after anesthesia or surgery. From the etiology, inflammation caused by surgery, central nervous system inflammation, glial cell activation, central nervous system oxidative stress, and the effect of oxidative stress on neuronal integrity and signal transduction are the causes of POCD. In addition, POCD is not a general consequence of surgery. Physical illness, weakness, low education level, and a history of alcohol or opioid or anticholinergic drug use may also occur. Anesthetics are also potential factors for the occurrence of POCD including sevoflurane. Older patients who underwent major surgery were more likely to develop POCD after sevoflurane inhalation than patients who received intravenous propofol.^43^ A growing number of studies have shown that sevoflurane may cause neuroinflammation and impair cognitive function, eventually resulting in POCD.^44^ Neuroinflammation is an important feature of many neurodegenerative diseases. This inflammatory response is mediated primarily by several mediators, many of which are produced by central nerve cells, including microglia and astrocytes. In addition, endothelial cells and perivascular macrophages are also important in releasing and transmitting these inflammatory signals in the central nervous system.^45^ For example, lipopolysaccharide binds to the TLR on the surface of microglia to activate a variety of signal transduction pathways, which ultimately leads to the activation of NF‐κB. The activation of NF‐κB mediates the production of many mediators that induce inflammation, such as iNOS and COX‐2, which jointly cause neuroinflammation.^46^ Studies have shown that the activation of NF‐κB can also mediate the abnormal activation of NLRP3 inflammasomes in microglia and release the proinflammatory factors IL‐1β and IL‐18 outside the cell to participate in the occurrence and development of a variety of neurological diseases.^15^ Sevoflurane activates microglia cells through activation of the NF‐κB signaling pathway and increases the release of inflammatory factors, such as IL‐1 and IL‐6, which damages neurons. It has been reported that the activation of microglia is increased in mice 1 week after surgery, and it causes inflammation in the hippocampus, which leads to the decline of cognitive function.^47^ Therefore, sevoflurane‐activated microglia also plays an important role in POCD.

The occurrence of neurodegenerative diseases may also be related to the disorder of microglia phagocytosis. Many microglia genes involved in phagocytosis are associated with AD and frontotemporal dementia^48^, ^49^ Polymorphism in the phagocytic receptor tyrosine kinase (MerTK) encoding gene is a risk factor for multiple sclerosis.^50^ Rett syndrome results from full mutation of the Mecp2 gene in microglia by reducing microglia phagocytic activity.^51^, ^52^ The B‐cell receptor CD22 is now considered to be a negative regulator of microglial phagocytosis in mice.^53^ Blocking CD22 restores microglial homeostasis in the aging brain and prevents age‐related cognitive decline, emphasizing the role of phagocytosis in clearing debris and maintaining neural function. It can be seen that the development of microglia is closely related to neurodegenerative diseases.

### 4.4 Summary and prospect

There is no denying that sevoflurane exposure is associated with neurodegeneration; however, there are study tips in cerebral ischemia‐reperfusion injury, traumatic brain injury, sevoflurane by inhibiting the activation of microglia, reduce rats tissue excessive oxidative stress and inflammation, and protection of injury of rats.^54^, ^55^ Does this suggest that sevoflurane can activate microglia to exert anti‐inflammatory effects and reduce brain damage in the existing brain tissue damage, while in normal brain tissue, exposure to sevoflurane leads to the accumulation of neurotoxic substances and mediates microglia are activated for a long time, increase the expression of inflammatory factors through various inflammatory signaling pathways, release inflammatory mediators to cause oxidative stress, damage nerve tissue, and eventually develop into neurodegenerative diseases.

**Figure 1:**
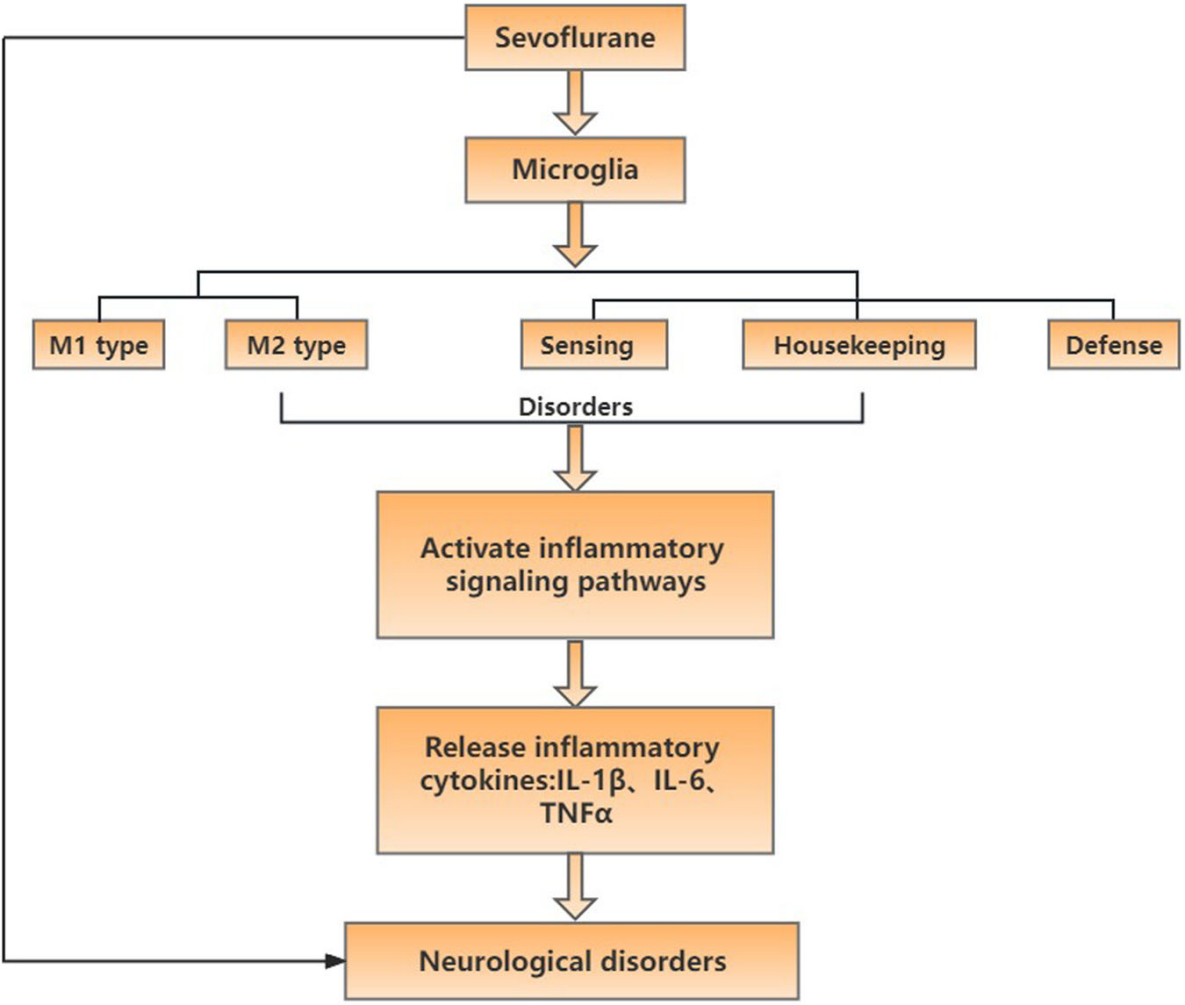
The flow chart of this article. Sevoflurane activated microglia to activate the inflammatory signaling pathway, releasing inflammatory cytokines interleukin (IL)‐1β, IL‐6, tumor necrosis factor (TNF)‐α, and mediating the occurrence of neurodegenerative diseases [Color figure can be viewed at wileyonlinelibrary.com]. [Correction added on 17 May 2024, after first online publication: This figure was revised in this version at the request of the authors.]

In summary, sevoflurane activates microglia and increases the expression of inflammatory factors through the inflammatory signaling pathway to injure or kill neurons, promoting the occurrence of related neurodegenerative diseases. Activated microglia showed a duality, that is, they had toxic or protective effects on neurons. Neurons may also be damaged when various functional links of microglia are damaged or maladjusted. Therefore, the understanding of sevoflurane on the expression process of microglia inflammation and the pathogenesis of related diseases (Figure 1) can provide multiple targets for the prevention and treatment of neurodegenerative diseases and is useful for the clinical treatment of neurodegenerative diseases.

## CONFLICT OF INTERESTS

The authors declare that there are no conflict of interests.

## ETHICS STATEMENT

Not applicable.

## AUTHOR CONTRIBUTIONS

Yan‐Li Huang collected material and composed the paper and Zhao‐Qiong Zhu modified the review.

## Data Availability

Data sharing not applicable to this article as no datasets were generated or analysed during the current study.
